# Bibliographical Notices

**Published:** 1856-01

**Authors:** 


### BIBLIOGRAPHICAL NOTICES.

*History of Medicine, from its origin to the nineteenth century, with an appendix, containing a philosophical and historical review of medicine to the present time.* By P. V. Renouard, M. D. *Translated from the French* by Cornelius G. Comegys, M. D., Professor of the Institutes of Medicine, Miami Medical College. Cincinnati: Moore, Wilstach, Keys & Co. 1856.

The study of history would little deserve the time and attention bestowed upon it, if it were confined to the bare knowledge of past transactions, and an inquiry into the eras when each of them happened. It concerns us but little to know that there were once such men as Alexander, Cæsar, Aristides and Plato, and that they lived in this or that period; that the empire of the Assyrians made way for that of the Babylonians, and the latter for the Medes and Persians, who were themselves subjected by the Macedonians, as these were afterwards by the Persians.

But it highly concerns us to know by what methods these empires were founded; by what steps they arose to that exalted pitch of grandeur which we so much admire; what it was that constituted their true glory, and what were the causes of their declension and fall; and we shall also do well to study the character and disposition, the talents, virtues, and even vices of those by whom they were governed, and whose good or bad qualities contributed to the grandeur or decay of the States over which they presided.

If, in these few words, we have indicated the true way to study general history, it then follows that the like method should be adopted in the study of special history, and we are convinced that it is the only plan of studying the history of medicine with profit and advantage.

It is not enough to know what system or theory preceded or followed the other, but we must go further, and see how and by whom these were founded; what were the causes of their advance; what the causes of their fall. Thus studied, medical history is of immense value to the student of medicine. We then draw from it such lessons of experience as will enable us to work out more successfully a nobler future—thus studied we shall also be able to realise the fact that it is as much written for our warning as for our guidance.

With these few introductory remarks, we beg to introduce to our readers M. Renouard's History of Medicine in an English dress. It is not our purpose to make a review of the work, for the space allotted us is altogether inadequate for such an undertaking. What we have to say, therefore, must necessarily be brief and unsatisfactory, but we could not allow so excellent and timely a publication to pass altogether unnoticed.

In 1846 M. Renouard published his *Histoire de Medicine* in 2 vols. In these he left untouched the first half of the present century. He, however, promised at the end of his preface, "to give hereafter, under the title of materials for contemporaneous medical history, a discussion of the theories, discoveries, and improvements that have signalised the first half of the 19th century." This promise he fulfilled in 1850, in a series of letters addressed to the editor of *l' Union Medicale.* These letters very appropriately form an appendix to the work now before us.

M. Renouard divides his subject into three great ages. The first includes the foundation of medical science, and extends from the mythological age to the time of Galen, or the termination of the second century of the Christian era. The second, which he terms the age of transition, includes the time between the end of the second century and the commencement of the fifteenth. The third is the age of renovation, and includes the time from the commencement of the fifteenth century to our own era. These ages he subdivides into periods or epochs. The first is the primitive period, or period of instinct, and ends at the fall of Troy, about twelve centuries before the coming of Christ. The second includes the mystic, or sacred period, and occupies from the fall of Troy to the dispersion of the Pythagoreans, 500 years before Christ. The third is named the philosophical period; it occupies about 180 years, and terminates with the foundation of the schools at Pergamos and Alexandria, 320 years before Christ. This school commenced the anatomical period, which ended with the second century after Christ. It was followed by the Greek period, (the fifth) which continued until the destruction of the Alexandrian library, in 640. Science then passed over to the Arabians, and the Arabian period commenced, to end with the fourteenth century. With the revival of letters medical science revived, and the erudite period began, which, so soon as the recovery of ancient literature was perfected, ended in the last, or the reformation period, and which includes the seventeenth and eighteenth centuries. Here, as we have already said, his history proper stops.

In taking a panoramic view of medical history, says an able writer, "we find it divided into two great periods, each in one degree the counterpart of the other. The one extends from the Greek origin of the science to the irruption of the Saracenic tribes, and is complete in itself, inasmuch as it comprises the first rise and extinction of medical science in Europe. Just as the knowledge of Asia and of Egypt was extinguished by passing over with empire to the Greeks, so the knowledge of the latter passed over to the victorious Arabians, and Europe sunk into barbaric darkness. This period continued until the age of renovation, which began with the fall of Constantinople, the immigration of learned Greeks into Italy, and the culminating point of Saracenic power in the fourteenth century. The second period is, however, as yet incomplete, inasmuch as the extreme point of development is not yet reached. In all other respects it may be compared with the first. It had this advantage, however, that it commenced on a wider basis than its analogue. The accumulated knowledge from which the science of the first period was developed was imperfect, because the sciential records of the mystic age antecedent to it were confined to an exclusive priesthood and hieroglyphical language, and much, therefore, of Asiatic and Egyptian learning was lost to the Greeks. They began science almost anew, and transmitted their accumulated knowledge to their successors in a living language, and a language most perfect and rich. It was also expounded to them by men of acute and polished minds, who read and spoke the language in which it was written. This was an immense advantage; it was the advantage which the student with a master possesses over the student without a master. Nevertheless, the powers which guided the development of medical science in the two periods, the obstacles which arose, and the methods which were adopted to overcome them were the same in both."—*Brit. and For. Med. Rev.*

We shall now introduce a few extracts from M. Renouard, showing his plan of procedure in the execution of his task, and the extensive and profound views which he takes of his subject.

"Celebrated physicians," he says, "influence the progress of their science and the value of their art, not by their writings only, but by their oral teachings, character, and conduct. Their lives offer often models for imitation, and sometimes, also, faults and errors to be avoided. Often, too, the early education of a man, and the circumstances in the midst of which he was reared, explain the peculiarity of his genius, and give the key to his successes and reverses. For these reasons I could not neglect entirely some biographical details relative to the most famous physicians, especially when these details had some connection with the general history of the art, or embraced some moral considerations.

"The sciences do not pursue their march isolated from each other, they go hand in hand, and it is rare that their progress is not simultaneous. An exception to this rule, however, presents itself in the history of the human mind in Europe. During the middle ages dialecties and theology are (were) cultivated successfully, while the other branches of human knowledge, and medicine in particular, merely vegetate (vegetated) in deep neglect. But with the fourteenth century, industry, the sciences, and the arts awake (awoke) from their long sleep.

"On the one hand, the civil and political organization of European nations becomes (became) regulated, their material good increases (increased); on the other, the intellectual and moral faculties of individuals are (were) developed, the mind makes (made) efforts freer, bolder, and in a better direction. The historian of medicine would fail, it seems to me, in one of his duties, if he did not now and then give a general view of the state of society. Therefore at the commencement of each of my chronological divisions I give a rapid sketch of the aspect which civilization then presented.

"Another fact extremely remarkable, and of capital interest in the history of medical theories is, that they are all derived more or less directly, from some system of philosophy, so that only an incomplete idea of them could be obtained if the philosophic sources from which they were drawn were unknown. But too much importance must not be attached to these analogies, nor must the value of medical theories be judged by them. It must not be forgotten that a philosophic system might be false as a whole, and yet true in its particular application to medicine. On the other hand, we may, by false logic, deduce an erroneous medical theory from an irreproachable philosophic system. Thus, then, after having indicated the philosophic ideas with which each medical doctrine may seem to be related, we shall judge this in itself, and relative to its practical consequences."—pp. xiv. xv.

With these extracts, we bring our very imperfect notice of

M. Renouard's history to a close. We trust that the book will be extensively read, as we are certain that a careful perusal of it will tend to dispel very erroneous opinions held, both in and out of the profession, on the subject of medical history. It will show, unless we are much mistaken, that the history of our noble calling presents in its progress, from its first dawn to the present time, as brilliant a career as that of any other branch of human learning—not even excepting Astronomy; that it has ever been moulded by the philosophy of the time; and that even in its darkest days it has had within itself sparks of vitality, which only required a better philosophy to enable them to blaze forth in their full glory.

One word more. Much has been said about the uncertainty of medical theories; and the different opinions of those who cultivate medicine are adduced as evidence of the low rank it holds as a science—as if the other branches of learning did not exhibit the very same features. Where, we may ask, is the certainty of the law, or political economy, or the laws which regulate trade? Nay, we may now go further, and ask, where is the *certainty* of religious teaching? Are Theologians as yet agreed among themselves upon the true exposition of many of the dogmas of their holy calling? Let the mass of controversial divinity, imbittered as it is in its every page, with the "*odium theologicum*," answer. Yet *these* have the certain word of God, from which to excogitate their theories, whilst *we* have to deal with the ever-changing phases of vital organism. All learning is uncertain, inasmuch as human reason is fallible, and we readily admit, to this extent, no more, the uncertainty of medicine.

But we strongly object to the argument, that because its followers are as yet agreed on no complete and incontrovertible theory, and because early opinions advanced on imperfect evidence, have yielded, in succession, to more extensive discoveries, therefore nothing certain is known upon the whole subject, and that all medical deductions must be crude, unauthentic and conjectural. We candidly admit that the time has not yet arrived when a perfect theory of medicine can be fixedly and finally established, since we have not yet before us all the facts on which such a theory may eventually be founded; but, in the meanwhile, we have abundant evidence of numerous and indisputable phenomena, each establishing important and undeniable conclusions; and the aggregate of these conclusions as they accumulate, will form the basis of future theories, each more and more approximating to perfection. Admitting, therefore, that we have yet much to learn, we contend that much sound knowledge has been already acquired, and we protest against the rejection of established parts, because the whole is not yet made perfect.

Dr. Comegys has executed his task very satisfactorily. We have noticed several trifling errors in his translation, the result, no doubt, of too great haste, but on the whole the text reads smoothly, and the author's meaning is clearly rendered. We sincerely trust his labors will meet with the success they so richly deserve.

---

*On Bandaging, and other Operations of Minor Surgery.* By F. W. Sargent, M.D., Member of the College of Physicians; one of the Surgeons to Wills Hospital, &c. &c. *New edition, revised and enlarged, with one hundred and eighty illustrations.* Philadelphia: Blanchard & Lea. 1856.

We are glad to see that another edition of this useful work has been called for. It shows a desire for a kind of knowledge which is too often deemed of no importance. The tendency which exists at present to substitute College Clinics for Hospital Surgery, necessarily sends a great many young men abroad who have never seen a fractured thigh or leg, and of course they are not familiar with the practical details of dressing. It is therefore of great importance to those who have never seen a fractured limb in these walking clinics, to have at their disposal a work like the present to complete, in some measure at least, so necessary a part of their education.

Dr. Sargent's work is the best of the kind, and we can recommend it in the strongest terms to all who may wish to refresh their knowledge of minor surgery.

The first edition, published in 1848, was highly commended by the press in this country and in England. The second edition contains many improvements and additions, which render it still more valuable and more in accordance with the present state of surgery.

In alluding to one of the novelties of the age—the treatment of Fracture of the Patella by adhesive strips—we do not think that the writer has done justice to Dr. Neill, whose paper on this subject was published in the January No., 1854, of this Journal.

After enumerating the various modes of treating this fracture, according to Cooper, Amesbury, Lonsdale, Boyer and others, he states that,

"M. Malgaigne, in his excellent 'Traité des Fractures,' p. 764, states that he has seen M. Gama, Surgeon to the Military Hospital of Val de Grace, treat successfully cases of transverse fracture of the patella, by means of strips of adhesive plaster passed above and below the fracture, in the form of the figure 8, the limb being placed upon the simple inclined plane. And in the Philadelphia Medical Examiner, p. 5, January, 1854, Dr. John Neill reports two cases of this injury in which this method was pursued. It is, undoubtedly, the simplest and the best plan which can be resorted to."

This certainly implies that Dr. Neill merely repeated a well known mode of dressing the fracture, according to the plan of M. Gama, and is, of course, entitled to no credit whatever.

The facts in the case are very different. Although Dr. N. in his paper claimed but little, and even suggested doubts as to its originality to the mind of the reader, by publishing Malgaigne's account of Gama's treatment, as well as Mr. Alcock's account of his use of adhesive strips; nevertheless, any one who has read the paper must admit, even if Neill's and Gama's modes of treatment are the same, that the former is entitled to the credit of originality, if not of priority. For, he expressly states that after treating these two cases, he then investigated the subject and found the statements which he quoted from Malgaigne and Alcock.

If Dr. N. had not published these quotations, we hardly think that he would have been said to have repeated or revived an old mode of treatment. Probably Dr. Sargent's attention was first called to Malgaigne by the quotation made, for the first edition of the Minor Surgery contains no reference to the subject, although published after Malgaigne's work had appeared. Dr. Sargent certainly knew that this treatment had not become general, although he states that "it is undoubtedly the simplest and the best plan which can be resorted to." He probably never saw or knew of a case treated in this way until Dr. Neill's publication was made, and therefore it is hardly fair to speak of Dr. Neill's paper as a report of "two cases of this injury, in which this method (Gama's) was pursued. The two modes of treatment are however *very different.*

M. Gama applied in the form of a *figure of* 8, *very long strips of adhesive plaster over graduated compresses.*

In the first place, Dr. N. uses no graduated compresses, but applies the plaster to the skin directly above and below the fragments, so as to make the plaster grasp them firmly, which cannot be done if a compress be interposed.

In the next place, Dr. N. does not use long strips applied in the form of a figure of 8. A figure of 8 around the knee is very much like a roller, for although the two rings of the 8 are above and below the patella, they are joined in the hams and compress the parts almost circularly; whereas, in Dr. Neill's dressing, the plaster is made to act as much as possible in the axis of the limb, i. e., the numerous strips applied to the lower fragment are directed upwards upon the thigh as nearly parallel with it as can be effected; and the strips which drag the upper fragment downwards are made to adhere to the leg as nearly parallel to it as possible. This is certainly no figure of 8 bandage, either in its composition or application.

It is possible that Dr. N. has laid himself open to this misinterpretation, by making so very prominent those allusions to other writers which may seem to destroy the originality of his dressing. Our readers, however, can decide that question for themselves.

We trust that the publishers will soon see the present edition exhausted.

### *Clinical Lectures on Surgery.* By M. Nelaton. *From notes taken by* Walter F. Atlee, M. D.

"Nulla est alia pro certo noscendi via, nisi quam plurimas et morborum, et dissectionum historias, tam aliorum proprias, collectas habere, et inter se comparare."—Morgagni *De sed. et caus. morb. lib.* 14.

Philadelphia: J. B. Lippincott & Co. 1855. 8vo. pp. 755.

This is quite a good looking book. It is well printed on excellent paper, and is altogether very respectably got up. Yet it has proved, on several accounts, an embarrassing one to us; and we doubt not that many other readers, with equally good intentions, will find it no less puzzling. The publishers have done their part handsomely, as usual, and the reporter, or compiler, or editor—we hardly know what to call him—gives ample evidence of commendable industry and enterprise in his share of the production; but, with the best disposition towards all the parties interested, we regret that it is impossible to congratulate any of them on the result which is now before us.

As an unauthorized account of another man's occasional lessons, the work belongs to a class which must necessarily be regarded with dissatisfaction in some quarters and with distrust everywhere. It is still more liable to suspicion, however, because it is not only an unauthorized report of extemporaneous hospital lectures, but an unauthorized translation from a foreign language in which these lectures were delivered. Nor is it simply and purely an American version of certain clinical sayings and doings of a distinguished French teacher, for it is filled in with the reporter's own descriptions of the operations performed, and with this latter's observations on the progress and terminations of many of the cases; and it is further enriched from time to time with his "additional" comments on the surgical pathology of some of the diseases which are previously discussed by the Parisian professor.

The preface informs us that the volume

"Contains the publication of notes taken, during a period of three years, 1851-52-53-54, from the remarks he [M. Nélaton] made upon cases to which attention was particularly directed. The course adopted in their arrangement has been, as a general rule, to class under the same head those in which the same pathological lesion existed, though in some few instances, when the great interest of the case lies in the diagnosis, this plan has been departed from, and the case has been classed with others, from the fact that it was *not* one of them.

"When any remarks have been made, in addition to those of M. Nélaton, they are so placed as to be at once distinguished. This has been done very rarely, in fact almost solely, in order to make the work, as respects surgical pathology, more complete, by stating the results of microscopical investigations. These results have been taken from the works of M. Charles Robin and of M. Lebert."

We can not understand from this preface whether the arrangement spoken of is M. Nélaton's or Dr. Atlee's, and whether the remarks of the former were made at the bed side or in the amphitheatre. From the text it may be inferred that the arrangement belongs to Dr. Atlee, and that the lessons were given sometimes in the wards, but more frequently, perhaps, in the lecture room. Nor is it as easy as he seems to imagine, to distinguish by their "place" the remarks of the commentator from those of his principal. We could discover no sign by which it was safe to recognize them, except what may be afforded in the remarks themselves—a kind of distinction which is not always flattering to the writer and never satisfactory to his reader.

We are not called upon to propose a proper title for such a conglomeration, as by Dr. Atlee's own admission, this book appears to be ; but we are bound to say that its present title of Clinical Lectures on Surgery by M. Nélaton is a serious misnomer. No one who examines the different chapters can fail to perceive, what is foreshadowed in the preface, that a great injustice is done not only to an able and deservedly popular European teacher, but to a large number of students and practitioners in this country, who may be induced to secure the offered nondescript interpretation, with its arrangements and accompaniments, as a bonâ fide course of clinical instruction emanating from that teacher.

Although Dr. Atlee's style is not free from gallicisms, and is too often careless and confused, we had sufficient faith in his fidelity and translating competency to have been thankful for his preparation of an unadulterated series of notes of M. Nélaton's cliniques, such as he appears to have taken during their progress, much as we doubt the propriety of such a publication. Nor should we complain of any new grouping of the cases, or of his descriptions of the operations and eventual results, provided it were all done without interfering with the original matter of the notes themselves, and so that the extra notes might be clearly distinguished from the rest; but in regard to all surgical pathology not presented by the genuine record, we should vastly prefer, in such a connection, M. Nélaton's own views as given in his different works and papers, to any re-hash of Robin and Lebert and other writers, no matter how authoritative or how well rendered by the commentator.

Notwithstanding these unfortunate drawbacks, however, we have been considerably interested in the clinical histories of which so large and so varied a collection is presented. Many of the cases are trivial and only in the way, others are very meagrely and inconclusively reported; and quite a large number are most provokingly, sometimes absurdly, cut short with the announcement that, either through the withdrawal of the operator, reporter or patient, nothing more was learned of the case.

Still, enough remains of substantially instructive material to make an abundantly large and desirable volume. Without attempting to discuss the principles and practice enunciated in the numerous chapters, or to express any opinion on them, we may note a topic here and there, and shall transfer a few paragraphs to our pages.

The following in relation to a case of malignant pustule of the lower eye-lid conveys a good lesson.

"To say that the swelling, in this case, was considerable, would give no idea of what it really was; the whole of the left side of the face, of the neck, anteriorly and posteriorly, and of the upper half of the chest, was swollen.

"Such a swelling, M. Nélaton said, was not extraordinary, and he related several cases to show this. When a surgeon of the Bureau Central, he was supplying the place of Bérard, at the Hospital, when a *chiffonnier* came to the consultation with his neck enormously swollen: it was thought to be a submaxillary phlegmon, and as the interior of the mouth was very red, it was thought it would open there. This was at 10 o'clock in the morning, and at 3 o'clock in the afternoon he had been sent for to see the patient, who was dying. Going in haste, he found that the swelling had increased, and the signs of asphyxia were so great that the trachea was opened to relieve the difficulty of breathing; in seven or eight hours afterwards the man died. Seeking a reason for the enormous swelling, and examining the beard, the long and neglected beard of a *chiffonnier*, he found a malignant pustule of the most marked character. If he had examined the patient, as he ought to have done, M. Nélaton said, this pustule would have been discovered and cauterized, and the man would have recovered. Upon another occasion, a surgeon informed him that he had lost a patient, twenty-four hours after his entrance to the hospital, from a panaris. When he went to examine the body, he found upon the wrist a magnificent malignant pustule, and the huge swelling extending from it.

"There is a disease which, at the commencement, is very simple, which belongs, equally with this other, to the charbonnous affections; in it, there is at first only swelling of the eyelids, without pustule. The physician must interrogate, and see if the patient has not been handling animals or animal matters; for, in order to give rise to this disease, it is not necessary that the animal be living, the dead matter preserves its properties. M. Nélaton has seen skins that preserved this property; one, the skin of a hare, after washing and much handling. This form of charbonnous disease was described by M. Bourgeois, a physician of Etampes, who calls it *malignant œdema of the eyelids.*

"In this case, the patient had been cauterized, as was said before, by nitric acid, that had extended itself over the cheek, and a very large destruction of tissue had taken place. It had been applied three times, on three successive days, in place of which the surgeon should have made use of the actual cautery, one good, energetic application of which would have been sufficient. The cauterization here, it was true, had saved the man's life, but it might have been done in a preferable way."—Pp. 39, 40.

A still more important warning may be gathered from the following case.

"December, 1853. A young woman, twenty-seven years of age, a child's nurse, in whom a phlegmonous abscess had spontaneously developed itself in the sub-hyoidean region. A tumefaction there was very apparent, red, œdematous, and yet firm to the touch; the firmness was that peculiar kind of firmness, that resistance to pressure, possessed by certain inflammations of the cellular tissue. This tumefaction extended from the hyoid bone all down the neck as far as the insertions of the sterno-cleido-mastoid muscle. If it had been situated in the subcutaneous cellular tissue, it would not have been thus limited; and besides, the patient had difficulty of respiration.

"It was evident, at once, that it was necessary to open this phlegmon. Chloroform was administered to put the patient under its influence, and the tissues were divided, layer by layer, to the extent of about an inch, in the median line, in the place where the operation of tracheotomy is usually performed. Quite a large quantity of pus came out of the opening, and the patient was notably relieved thereby.

"M. Nélaton said it might be supposed that this patient would soon be cured, but it would not be so; she would be forced to remain a long time in the hospital, to say the least. His reason for saying so was that, upon opening the incision and examining the bottom of the wound, it was seen to pass between the muscles, into the cellular tissue in contact with the trachea. With a probe the peculiar resistance of the cartilaginous rings could be felt, and, in fact, the rings could be seen. Moreover, the probe showed that the cavity formed by the abscess was very extensive. Now it would be very difficult for these walls to unite, on account of the mobility of the trachea, and the peculiar vitality of the parts situated posteriorly; for if fleshy granulations are easily developed on cellular tissue or on bone, it is not so for fibro-cartilage; they have never been seen to form upon it. It was to be feared, therefore, that the patient would preserve the fistula for a long time.

"Abscesses formed in this situation must be opened early and freely, and in the direction of the greatest *décollement*, or separation of parts; for it is to be feared that the pus will proceed downwards further and further, even into the mediastinum. The danger of this is at once seen, by reflecting upon the anatomy of the region. The only means of preventing this is to make such an incision.

"In making the incision, the tissues should be divided layer by layer, as in the operation for tracheotomy. M. Nélaton related a case to show that the subclavian vein might be opened in these operations, or in operations in this neighborhood. He was sent for once to visit a woman living upon the island of St. Louis (a part of Paris, about one and a half miles from his residence.) She was a corpse when he arrived. The history of the case was, that the patient had a small tumor in the neck, which her physician had persuaded her to have removed; and, in performing the operation, a hemorrhage occurred that could not be arrested. In making an examination of the body, a hole was found in the subclavian vein, into which M. Nélaton passed his fore-finger. The tumor itself was an appendix of the thyroid gland. It is said, moreover, that the brachio-cephalic arterial trunk was once opened by a young student, who was hastening to open the trachea of a friend who was drowned, in order to make artificial respiration.

"The fistulous orifice in the place where the opening had been made in the abscess, was still discharging a small quantity of pus, when the patient left the wards, three weeks after the operation."—Pp. 123, 124.

In a case of abscesses over the sternum the annexed remarks are interesting.

"As an affection of the kind, existing in this instance, might be supposed to be caused by scrofula, several questions were put to the man, in order to learn something about it. He was rather subject to taking cold. Three years before, he had spit blood, but he could give no details, so that it could not be known whether it had proceeded from the lungs or not; no symptoms of any pulmonary affection could then be detected. He had become thinner, it was true, but the affection for which he had entered was quite sufficient to account for this; and, besides, his health was quite good.

"All these particulars were entered into, because an important question was to be decided, namely: Would it be proper to perform an operation for the relief of this patient? The affection was serious, a caries of the sternum, with denudation of the bone, suppuration, and no tendency to cicatrization. The suppuration was quite abundant.

The man was forced to work in order to live, so that, unless something were done to cure him, he must pass his existence in a hospital. The two abscesses then existing would be opened, and the bone could then be well examined, and the extent of the disease decided; perhaps the necrosed portion would be found ready to be eliminated. If a large extent of the sternum were affected, what was to be done? should a part of the sternum be excised? This is an operation of high antiquity; Boyer performed it once, but the surgeons of the present day no longer practise it. In fact, in all the resections of the sternum or of the ribs, that he had witnessed, M. Nélaton said he had always seen the patients die. They were worn out by the suppuration, or perished from putrid infection; for the parts are most unfavorably placed for cicatrization. But, in this instance, it really seemed as if such an operation ought to be performed; for the patient was young, and, without such relief, his condition would be excessively miserable.

"Both the abscesses were opened, but no denuded bone could be found, though the finger was introduced in every direction. The next day, a most careful examination, with a probe, was made with the same result. M. Nélaton, however, said that the not finding any was no decided proof that none existed. The patient continued in the wards, under observation, for about a week, and then left, in about the same condition as when he entered."—Pp. 128, 129.

Again, in relation to fracture of the patella:

"May, 1853. A young man, with a fracture of the patella from direct violence to the front of the knee. There was very considerable effusion into the articulation. The fracture was transverse; the separation of the fragments was but slight.

"After waiting until the swelling of the parts had entirely disappeared, the leg was placed in extension, and then the parts having been previously oiled, *as quickly as possible* bandages soaked in plaster* were applied. The bandages were hard in seven or eight minutes, during which time the fragments of bone were held together by the fingers (previously oiled).

"M. Nélaton said that he had used, several times, the instrument of M. Malgaigne, in order to keep the fragments of the fractured patella together, and that he had never found any of those inconveniences to result from its use, which have been so singularly exaggerated. This instrument is a double tenaculum, one hooked extremity of which is fastened into the tendon above the bone, and the other into the ligament below; by means of a screw, these two extremities can be brought towards each other and then fixed.

"At the expiration of thirty-five days, slight movements of flexion were made at the joint; every day, these movements were increased, and, some time afterward, the patient was allowed to walk. In this case, in all probability, there was bony union between the fragments."—Pp. 170, 171.

*"The mixture used was composed of equal parts of warm starch and of very dry plaster of Paris. It has the advantage of becoming solid almost instantaneously."

Also, a case of sprain as diagnosticated from a fracture of the fibula.

"December, 1852. A young girl, with signs of violence about the right ankle-joint. She had made a misstep, and twisted the foot. The question was, whether there was a sprain, or a fracture of the fibula. In the first place, there was no deformity, which renders the existence of a fracture evident, in some cases; here, there was only swelling. As regards ecchymosis, it existed on the dorsal surface of the foot, with two points more marked than others; one just below the external malleolus, the other above, along the course of the peroneal muscles. As regards local pain, there was nothing very characteristic; there was no one point where the pain was much more acute than at another. Another means of exploration, of great importance, above all when chloroform is administered to the patient, is the *ballottement* (*shaking about*) of the articulation. The fracture of the lower extremity of the fibula compromises the solidity of the joint, and there is lateral ballottement; the astragalus can be pushed from one side to the other. In order to detect it, the heel must be grasped as near to the articulation as possible, and then, if the muscles be relaxed, there will be ballottement, and even *clapottement* (*splashing*,) in some cases. This is a most excellent sign of this fracture. Again, the malleolus of the tibia appears to become more projecting, when the heel is pressed to the opposite side.

"In this case, there were none of these signs. As, perhaps, they were absent on account of the contraction of the muscles, chloroform was administered, but still they were not to be detected. There was, therefore, no longer any doubt; there was no fracture.

"As to the ecchymosis in this case, it favored the idea of a sprain, being about the heel, below the articulation, where the ligaments are, and above it, along the peroneal muscles. To explain this last point, M. Bonnet, of Lyons, has noticed that when the fibres of the ligaments are torn, some of the fibres of the muscles are ruptured also."—Pp. 180, 181.

The operation for obliteration, by actual cautery, of the lachrymal sac in obstinate fistula, is pretty fully presented in the subjoined extract.

"Here, M. Nélaton said, he was very much inclined to make use of cauterization. This method was used in the most ancient times; but the ancients considered lachrymal tumor to be but a simple ordinary abscess. Fallopius was the first who discovered the lachrymal canal; Galen had seen the lachrymal puncta, but he imagined them to be orifices, through which glands poured a secretion of tears upon the surface of the eye. One century later, Maistre Jean, a Frenchman, first thought of the connection between the pathology of lachrymal fistula and the anatomical structure of that region. Some time after him, Anel used injections in the treatment of these cases, and cauterizations were abandoned, all the attention of surgeons being directed to the restoration of the natural passages. It is only of late years that the ancient method has been revived again, by Desmarres, Sichel, and others. In old times, the design of the surgeon in performing this cauterization, was to destroy carious bone, by which it was thought that the fistula was maintained; nowadays, however, it is done with the object of producing the obliteration of the lachrymal passages. That which the surgeon did formerly without being aware of it, he does at the present day from design.

"In these cases of fistula lachrymalis, it should be remembered that there are two things, for which the surgeon operates; one is the overflowing of the tears, the other is the pathological condition of the lachrymal sac. It may happen that the canal is permeable, and yet its walls secrete pus.

"M. Desmarres, who has paid great attention to this operation, takes most particular care not to cauterize the skin. He lays bare the sac by an incision, of quite considerable extent, four-fifths of an inch in length, commencing above the tendon of the orbicularis muscle, and dividing it and the skin in a direction from above downward, and from within outward. This being done, the two lips of the wound are separated; this is a delicate step of the operation; the section of the nasal arteries must be avoided as much as possible. The two flaps are held apart by two instruments, a kind of small rake invented for that purpose, and a compress is applied over the eye for its protection. The next step of the operation is the application of the cautery, and to do this well, there are some things with which the surgeon must be acquainted. The lachrymal sac is not globular, as might be supposed, but it extends backwards; there is an *arrière-cavité*, owing to its being bound down in front. The operator should always place himself on the same side as the fistula, so that, on no account, he may turn the point of his instrument towards the eye. It is a good plan, also, to tampon the sac, after it has been opened for fifteen minutes, so as to apply the cautery to a dry surface.

"It has been objected to this operation, that the lachrymal passages being obliterated, the tears must necessarily flow over the cheek. But many cases are recorded, in science, of their obliteration by accidents, without epiphora being the result; and the fact is, that epiphora shows itself very rarely after the cauterization. The eye, no longer irritated by the fistula in its neighborhood, the tears are secreted in less abundance; and it may be that the secretion is diminished by the destruction of these ducts. Moreover, M. Nélaton believes that a great portion of the lachrymal secretion always disappears by evaporation.

"The operation was performed as described above. The next day an erysipelatous inflammation declared itself, which lasted for some days. Notwithstanding this complication, at the expiration of twelve days the wound was almost completely cicatrized; there was no pain, no swelling, and the patient said she was not annoyed by lachrymation; there was no evidence of epiphora. About ten days afterward she left the hospital, believing herself to be cured; and M. Nélaton said that he *thought*

that she was; he evidently, however, had some doubts about it, and asked her to come back at once, if she had any return of the symptoms. He also called the attention of the class to the fact that, by pressing upon the upper lachrymal duct, some mucus issued from the puncture, showing that the obliteration was not complete.

"This patient never returned to the hospital, so that, very probably, the cure was a permanent one."*—Pp. 398, 400.

One more quotation, and we must close without further remark. In relation to bloody tumor of the pelvis, we find the following interesting matter under the head of "Retro-Uterine Hæmatocele."

"December, 1853. A young woman, who had become pregnant fourteen months before her entrance, but aborted without any known cause, at the fifth month. Everything went off very well; she said that, since then, she had suffered from headache; but she mentioned no symptom having anything to do with the affection at present under consideration. She had been for some time very irregular in her menstruation, both as regards time and quantity; for the last four months she had seen nothing, and entered the hospital on account of suffering a great deal at the genital organs.

"The fingers in the vagina encountered the neck of the uterus not very far from the vulva, and carried forwards; it was small, as in women who have had no children, with a small orifice, and flattened antero-posteriorly. Almost directly behind it was a mass, and as the posterior wall of the vagina is soft and flexible, you could, by pressing, appreciate the projection of the tumor. In the hypogastric region was a mass, in which was a depression, like the top of the heart of playing-cards, when movements were given to the tumor in the vagina, they were communicated to the right portion of the tumor, and not to the left. This tumor was evidently not uterine, but was placed against and behind the uterus. It was either a retro-uterine hæmatocele, or a phlegmon of the ovary; it was not an extra-uterine pregnancy, for in such cases the uterus undergoes changes. The consistence of the mass was *pasty*, not evidently fluctuating; it had been rapidly developed, and was regular in form: it was placed behind the uterus, and that organ had its neck thrown forward behind the symphysis; M. Nélaton, therefore, decided it to be a hæmatocele. Moreover, the tumor was observed in a young woman who had trouble in menstruating; and again, there was one more symptom of its being a bloody tumor—there was a bluish tint, a bluish trans-

*"The fact mentioned in the above case, of the flow of mucus from the puncture, is interesting; for Delpech, the celebrated surgeon of Montpellier, who frequently had recourse to obliteration of the lachrymal sac, says that the cases of cure are those in which the action of the caustics is limited to making disappear the inflammation of the lachrymal passages, without producing their obliteration, whilst the unsuccessful cases depend precisely upon the fact of the obliteration being complete."

parency of the vagina covering it; its thin wall permitted the color of the blood to be seen behind it. There was a small irregularity in the position, in this case, that has been observed before, namely, that above, it was not placed behind the uterus, but at the side of it.

"These tumors must be quite common. Four years before, at furthest, one of M. Nélaton's *internes* published some cases, and, soon afterward, all of the surgeons of the Parisian hospitals reported others. There is something strange in the affection; it is never found in the dissecting-rooms. This is owing, however, to the fact, that the affection is but an effusion of blood, and it is gradually absorbed. He referred to the case that had been in the wards some months before, when the affection had been supposed to be an ovarian tumor fallen into the cavity of the pelvis; not thinking this diagnosis to be correct, the patient had been kept in the wards, and at the end of several months there was not a trace of the tumor. This, the absorption of the blood, is one of the modes of termination, and the most happy; at other times, the rectum is perforated; at others, the vagina. These other terminations are sometimes fortunate also; yet they have their dangers, for putrid decomposition has been seen to occur, and some of the patients have died. M. Nélaton has seen one such case in the wards of M. Marotte, and another in those of M. Moreau. On this account, therefore, the danger of putrid infection, M. Nélaton, now-a-days, takes good care not to open the sac; not from what he had seen in his own practice, for all had recovered, but from what he has witnessed in others. The opening should never be made; you can always rely upon the absorption of the mass.

"This patient remained in the wards until the end of January, gradually getting better; she then left the hospital; as the cure would be as well completed at home.

"March, 1854. A young woman entered the wards, and said that, four or five months previously, she had commenced to lose blood in the urine; this hæmaturia, after having lasted for some time, had ceased, and it had been replaced by a loss of blood from the uterus. This uterine hemorrhage was still continuing when she came into the wards.

"Upon examination above the pubis, a pelvic tumor was found, placed behind the uterus, towards the left side. The finger being introduced into the vagina, the tumor could be moved, and felt to be distinct from the uterus; its consistence was peculiar—it was like paste. The neck of the uterus in this patient was directed backward, which is an exception to the general rule in these cases; the cause of it was not known. Just behind the neck, the lower part of a globular mass was distinctly to be felt. M. Nélaton was of opinion that this was one of those *bloody tumors*, and that the proper mode of treatment was temporization.

"M. Nélaton said that, now, his *education was made* as regards these tumors. The first case he had seen, he had sent away as incurable; the second case (it came the same week as the other) he thought about a long time, and at last determined to introduce a trocar; it was either an encephaloid mass, a cyst, or an abscess; if the first, the puncture could do no harm, for the patient must die; and if a cyst or an abscess it would be emptied. Very much to his astonishment, the introduction of the trocar gave rise to a discharge of a pint of liquid, of the color of molasses. Another case came into his hand soon afterward; a woman who had hemorrhage, and a large tumor having the same position; a trocar was introduced, and the opening afterwards enlarged; two pints of liquid, black and with dark clots, came out. Formidable symptoms followed this opening: the sac inflamed, and the patient's situation was very critical for some time; but she eventually recovered.

"Just at this time, a patient went to M. Malgaigne; he thought it an interstitial fibrous tumor of the uterus. At that time, it was the practice to make incisions and extract these polypi; he, therefore, cut right and left; but when the limits of the neck of the uterus were passed, to his great astonishment, he entered a large sac of blood. This patient died in four days; at the autopsy, the ovary was found projecting, with bleeding points, into the sac. Soon after this case, another came to M. Nélaton, and he opened it, and extracted the contents. Very serious symptoms took place; but after being in great danger, and causing great alarm, she recovered. Seeing the dangers of opening these tumors, he asked himself if these spontaneous effusions would not conduct themselves here as elsewhere. In other parts of the body they are absorbed; and why should they not be absorbed in this situation as well as in any other? Soon afterward, a patient, with a large tumor, came under his care; he waited, and at the end of four and a half months all had gone. Since then, a great number of singular facts have been accumulated. About two years before, a patient had been in the wards—the same whose case is first reported—when most competent surgeons had diagnosed the affection to be either an abscess or an ovarian tumor; M. Nélaton, however, thought it a retro-uterine hæmatocele, and in the course of time all disappeared; he had seen this patient but a few days before, and she was perfectly well. They sometimes terminate in other ways, and spontaneous cure sometimes results from their opening into the vagina and rectum; this spontaneous opening, moreover, is less dangerous than the artificial. In 1837, this affection was described for the first time, and already thirty cases have been reported by the surgeons of Paris.

"After being a few days in the wards, this patient commenced to lose by the vagina a very dark liquid, and while doing so, the tumor notably and proportionately diminished. At the upper part of the vagina was found a perforation, whence the contents of the tumor were discharged. In a few days, the patient felt so well she was unwilling to remain longer in the wards."* Pp. 711–714.

* A discussion on these sanguineous pelvic cysts, in the Medical Society of London, is to be seen in the second volume of *The Lancet,* for 1852, p. 553.

*An Introduction to Practical Pharmacy: designed as a Text Book for the Student, and as a Guide to the Physician and Pharmaceutist. With many formulas and prescriptions.* By EDWARD PARRISH, Principal of the School of Practical Pharmacy, Philadelphia. *With two hundred and forty-three illustrations.* Philadelphia: Blanchard & Lea. 1856.

It is very seldom that a subject like pharmacy, so entirely practical and necessarily devoid of much originality, can be so treated as to form a book alike valuable for its possession of new and useful information, and for the judicious selection and arrangement of the formulæ and directions required both by the novice and the adept in the daily practice of their profession. We therefore sincerely congratulate the author in having succeeded in producing a work which we think will be found ere long by the side of the U. S. Dispensatory and the U. S. Pharmacopœia, upon the desk of nearly every pharmaceutist in our country. Mr. Parrish states in his preface, that as a teacher of pharmacy, he has "long since experienced the want of a book which should contain the leading facts and principles of the science arranged for study, and with especial reference to those features of the subject which possess a practical interest to the physician." And it is to the medical student at the commencement of his studies to whom we particularly recommend his book, as a most useful guide not only in acquiring a practical knowledge of this study, but also in his subsequent career as a practising physician.

A description of the apparatus and fixtures necessary in the dispensing and compounding room is first given, with numerous engravings, illustrating the various forms of bottles, jars, gallipots, scales, weights, measures, mortars, pill machines, funnels, displacement apparatus, etc.; these form the contents of the first chapter, and contain many details regarding the use and varied forms of the several articles, the value of which will be felt by all who possess any experience on this point. The second chapter contains tables and directions for converting the different systems of weights into each other; tables by Messrs. Durand, Procter and the author, showing the number of drops of various liquids in a fluid drachm; tables and instructions for taking specific gravities, and for converting the degrees of different hydrometers into their several specific gravities, the use of urinometers, etc. The next chapter is explanatory of the Pharmacopœia and of its system of nomenclature, its variation from the London and other Pharmacopœas, etc. This closes the first or preliminary part of the work.

The next division of the work (Part 2d) is devoted to "Galenical Pharmacy," consisting of directions for the proper collection and desiccation of medicinal plants, for the operations of solution, filtration, maceration and the preparation of infusions, tinctures, medicated wines and vinegars, and the officinal and unofficinal preparations of opium. This portion of the work also contains many useful suggestions for the various applications of heat necessary in the numerous processes of pharmacy. Some of the contrivances are new to us, and the whole chapter must prove most valuable to the student. The preparation of extracts, syrups, conserves, lozenges, etc., with a chapter devoted entirely to distillation and the utensils required therein, comprises the balance of this division. Part 3d consists of the history of the pharmaceutical plants and the products derived therefrom that are made use of by the apothecary. Part 4th, of the inorganic pharmaceutical preparations, the two forming together a synopsis of organic and inorganic chemistry so far as is essential to the proper understanding and preparations of the various remedies that the apothecary is obliged to prepare himself, or which circumstances may prevent him from obtaining from the manufacturing chemist. In part 5th the extemporaneous preparation of prescriptions and other medicines is treated of at length, and every direction given that is required to enable the student to supply the deficiency resulting from want of actual practice behind the counter. The formulæ for numerous preparations, not officinal but in general family use, besides being constantly prescribed by the physician, are here arranged under their appropriate heads, and must render the book, we think, useful to every practitioner.

We regret that our limited space prevents a more detailed analysis of the work, we must therefore content ourselves with referring the practising physician as well as the student in medicine or pharmacy to the work itself, believing that it will prove a most useful companion to every person who either prescribes or compounds medicines.

---

